# Spray-coated nanoscale conductive patterns based on *in situ* sintered silver nanoparticle inks

**DOI:** 10.1186/1556-276X-9-145

**Published:** 2014-03-25

**Authors:** Yifan Zheng, Shuguang Li, Wei Shi, Junsheng Yu

**Affiliations:** 1 State Key Laboratory of Electronic Thin Films and Integrated Devices, School of Optoelectronic Information, University of Electronic Science and Technology of China (UESTC), Chengdu 610054, People's Republic of China

**Keywords:** Spray coating, Conductive pattern, Silver nanoparticle ink, Inverted polymer solar cell, *In situ* sintering process

## Abstract

Nanoscale patterns with high conductivity based on silver nanoparticle inks were fabricated using spray coating method. Through optimizing the solution content and spray operation, accurate nanoscale patterns consisting of silver nanoparticles with a square resistance lower than 1 Ω /cm^2^ were obtained. By incorporating *in situ* sintering to substitute the general post sintering process, the time consumption could be significantly reduced to one sixth, qualifying it for large-scale and cost-effective fabrication of printed electronics. To testify the application of spray-coated silver nanoparticle inks, an inverted polymer solar cell was also fabricated, which exhibited a power conversion efficiency of 2.76%.

## Background

Printed electronics constitute an emerging class of materials with potential application in flexible devices including organic light-emitting diodes [1,2], organic thin film transistors [3-5], flexible and conformal antenna arrays [6], photovoltaic devices [7-10], radio-frequency identification [11,12], electronic circuits fabricated in clothing [13], and biomedical devices [14]. Recently, the exploration of silver nanoparticle inks has yielded a promising potential for the design of nanoscale conductive patterns for integration on plastic, textile, and paper substrates, which is compatible with the high-throughput and cost-effective fabrication of printed electronics.

Among the conventional pattern technologies of printed electronics based on silver nanoparticle inks, inkjet printing is the most widely applied due to its great potential for a variety of substrates as well as high-throughput and cost-effective system. Silver nanoparticle inks were directly ejected from the nozzle to the substrate and then sintered at about 140°C ~ 250°C for 5 min to form final conductive patterns [15-17]. Silver nanoparticle inks based on inkjet printing are still hampered from practical application due to the reasons below. Firstly, solution properties including ink viscosity, surface tension, and solubility have a significant influence on the preparation of printed patterns [18]. The complex film preparation and drying process for different types of silver nanoparticle inks restrict their potential for reproducible conductive patterns [19,20]. Secondly, the coalescence of subsequently ejected ink droplets would cause edges in a type of wave rather than a straight line. Although this phenomenon could be modified by adjusting the component of the solvent, the wave-like edge is hard to avoid which would be even worse accompanied with the patterns at the nanometer scale, leading to conduction between the adjacent lines detrimental to the device [21]. Besides, both the low printing speed of inkjet printing and general time-consuming post sintering process hinder the potential of silver nanoparticle inks for the cost-effective fabrication of printed electronics [22].

Alternatively, emerged as a promising method, spray coating has been successfully applied in printing electronics [23,24]. Compared to inkjet printing, spray coating exhibits higher printing speed and easier control of the deposited film morphology [25]. However, there are only a few reports about spray-coated conductive patterns based on silver nanoparticle inks until now [22,26]. Therefore, in this work, the influence of spray coating silver nanoparticle inks on the properties of silver nanoscale conductive patterns was studied, and the morphology of the conductive patterns was characterized and analyzed by scanning electron microscopy (SEM) and electronic dispersive spectrometry (EDS) in detail. Also, based on the obtained silver nanoscale conductive patterns, polymer solar cells were fabricated using spray coating method, and the performance of the solar cells was also investigated.

## Methods

**Figure 1:**
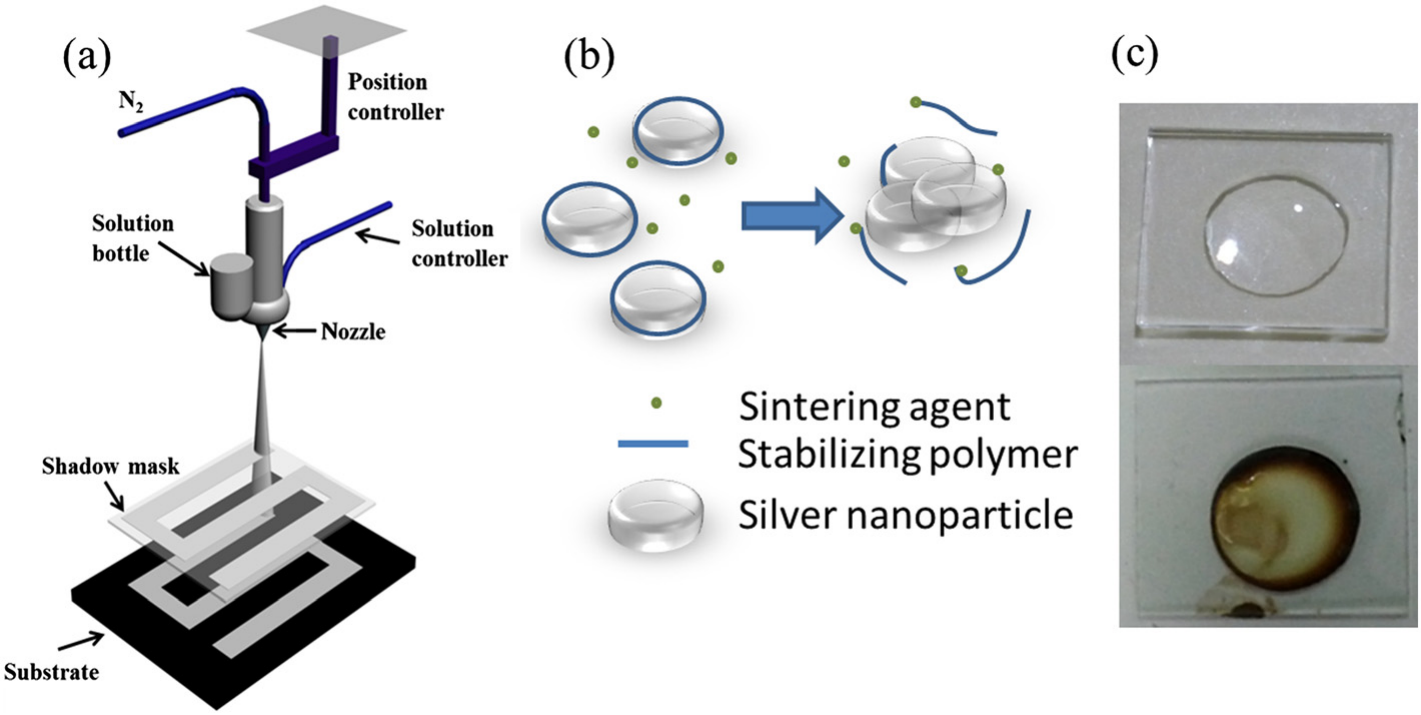
**Spray coating apparatus, sintering process, and coffee ring effect. (a)** Schematic diagram of the spray coating apparatus in this study. **(b)** Illustration of the sintering process of silver nanoparticle inks. **(c)** Image of the coffee ring effect on silver nanoparticle inks during the sintering process.

The device fabrication apparatus is shown in Figure 1a. The silver nanoparticle inks in solution were kept in a bottle and then sprayed directly onto the substrate under the pressure of nitrogen [27]. The shadow mask was utilized for patterning the image on the substrate, which was settled on the heater band for *in situ* annealing during the spray coating process. For the polymer solar cell (PSC) fabrication, the device configuration is indium tin oxide (ITO)/ZnO (40 nm)/poly(3-hexylthiophene) (P3HT)/ [6]-phenyl-C_61_-butyric acid methyl ester (PC_61_BM) (200 ± 15 nm)/PEDOT:PSS (30 nm)/spray-coated Ag [28-30]. ITO-coated glass substrates with a sheet resistance of 10 Ω/sq were consecutively cleaned in an ultrasonic bath containing detergent, acetone, deionized water, and ethanol for 10 min each step and then dried by nitrogen blow. Prior to the deposition of functional layers, the substrate was treated by UV light for 10 min. The ZnO precursor was prepared by dissolving zinc acetate dihydrate (Zn(CH_3_COO)_2_ · 2H_2_O, 99.9%, 1 g, Aldrich, St. Louis, MO, USA) and ethanolamine (NH_2_CH_2_CH_2_OH, 99.5%, 0.28 g, Aldrich) in 2-methoxyethanol (CH_3_OCH_2_CH_2_OH, 99.8%, 10 ml, Aldrich) under vigorous stirring for 12 h for the hydrolysis reaction in air. A 30-nm ZnO ETL was spin-cast from the precursor solution on top of the clean ITO-glass substrate and then annealed at 200°C for 1 h in air [31]. Next, an active layer consisting of 1:1 mixture of P3HT (99.9%, Aldrich) and PC_61_BM (99.9%, Lumtec, Mentor, OH, USA) was prepared in 1,2-dichlorobenzene (DCB) at a concentration of 4 mg/ml and then spray-coated at a rate of 0.30 ml/min at a height of 20 cm. PEDOT:PSS was sprayed at a rate of 0.35 ml/min at a height of 18 cm. The post annealing process was employed for modifying the active layer and PEDOT:PSS, which was at 140°C for 5 min and at 130°C for 20 min, respectively.

Throughout the whole PSC spray coating process, the airbrush was powered by N_2_ gas at a high pressure of approximately 60 psi to ensure a fine nebulization of solution.

The morphology of the nanoscale conductive pattern was characterized by SEM (JSM-6610LV) and metallurgical microscopy (Olympus BX41, Shinjuku-ku, Japan). The component of the pattern was analyzed by EDS (Oxford Instruments, Abingdon, UK). Current density-voltage (*J*-*V*) curves under illumination were measured with a Keithley 4200 programmable voltage–current source (Cleveland, OH, USA). A xenon lamp (CHF-XM35, Beijing Trusttech, Beijing, China) with an illumination power of 100 mW/cm^2^ was used as an illumination source. The thicknesses of the film obtained from the solution process were measured with a stylus profiler (Dektak 150 stylus profiler, New York, USA). All the measurements were carried out in air at ambient circumstance without device encapsulation.

## Results and discussion

Figure 1b illustrates the mechanism of the sintering process of silver nanoparticle inks, in which the stabilizing polymer is removed from the Ag nanoparticle surface upon drying the dispersion [32]. The coffee ring effect and Marangoni flow are important factors to determine the morphology of the resulting film during the sintering process [33,34]. As shown in Figure 1c, the solute would accumulate at the rim of a drying droplet under the influence of a surface tension gradient - the so-called Marangoni flow. In order to gain control over the homogeneity of the spray-coated film, we increased the vapor pressure around the drying feature by incorporating ethanol. The spreading capability according to the Marangoni velocity is

(1) $$v_{c}^{2}\left(x\right)=\frac{1}{2\eta \left(x\right)}\frac{\mathrm{d\gamma}}{\mathrm{dx}}x\left(1-x\right)\left(-A_{l}\alpha_{l}+A_{h}\alpha_{h}\right)$$

where *η* is the viscosity of the film, *γ* the surface tension, *x* the volume fraction of the low surface tension solvent, *A*_l_ and *A*_h_ the evaporation velocity, and *α*_l_ and *α*_h_ the activity coefficient of the low and high surface tension solvent, respectively [35]. Through optimizing the content of silver nanoparticle inks, it was found that 45 vol. % ethanol could not only reduce the contact angle of the mixture but also balance the Marangoni flow and convective flows in a drying droplet, resulting in the relatively homogeneous solid film.

**Figure 2:**
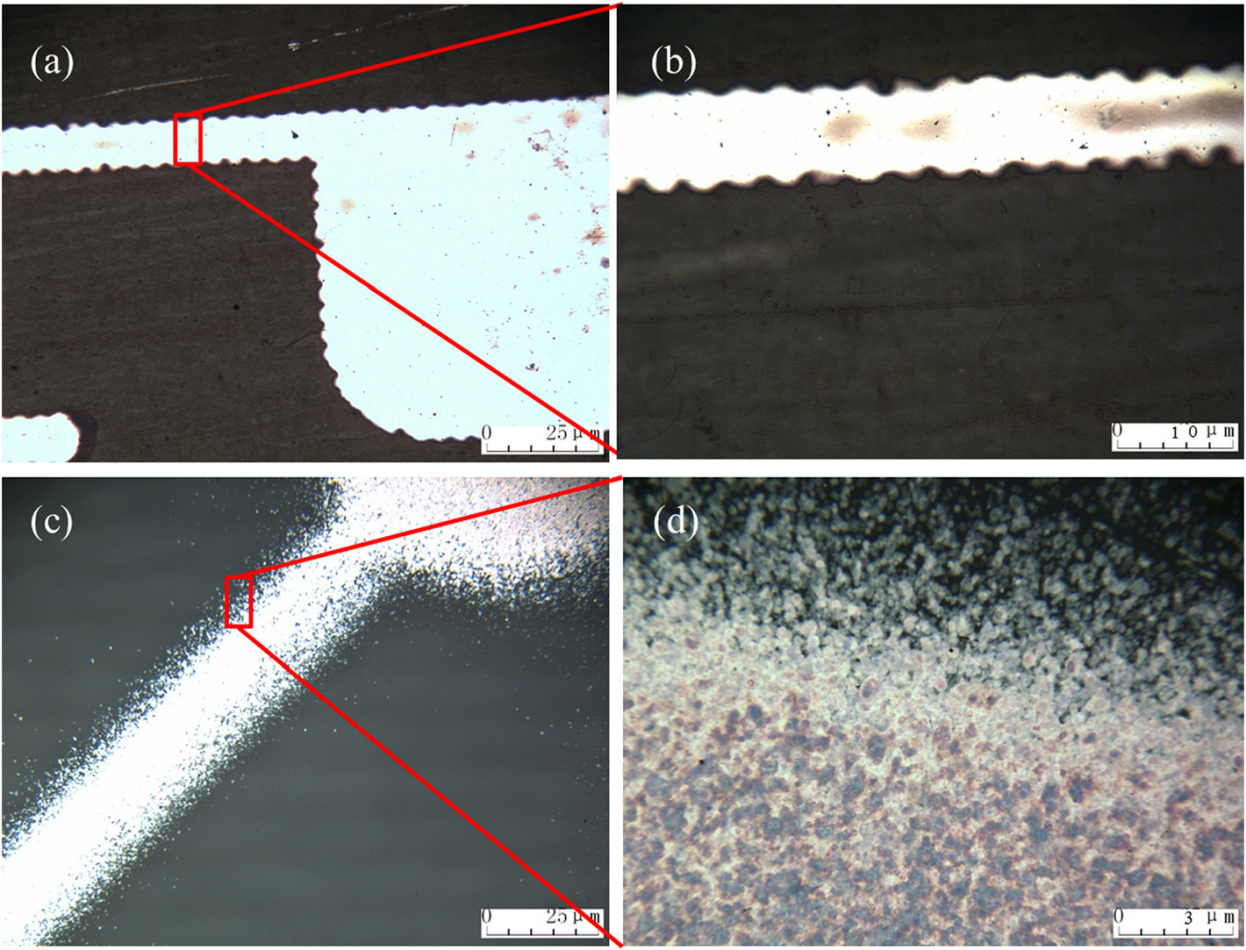
Metallurgical microscope images of the rim of the inkjet-printed (a, b) and spray-coated (c, d) conductive silver patterns.

Another factor that should be taken into consideration is the droplet size, which is mainly affected by the flow rate and gas pressure. Kim et al. [36] investigated the influence of spray condition on the droplet size and found that the sprayed droplet size would decrease with increasing gas pressure. The relationship between droplet size and spray height is depicted by the formula

(2) $$D_{\mathrm{av}}=\frac{\mathrm{W\lambda}}{2\pi k_{d}\mathrm{\Delta T}}$$

where *D*_av_ is the average droplet diameter, *W* is the average drying rate of the droplet, *λ* is the latent heat of vaporization, *k*_d_ is the thermal conductivity of the liquid droplet, and *∆T* is the mean temperature difference between the droplet surface and the surrounding air [37]. To avoid the diffraction of the sprayed droplet on the pattern, spray height should be set lower than 10 cm. However, a droplet of large size (>30 μm) would be formed in this situation, which may in turn result in large time consumption for film drying. Meanwhile, the overlapping between several droplets could lead to a rough surface and insufficient sintering of silver nanoparticle inks. In this case, decreasing the flow rate below 1.1 ml/min was necessary to obtain the droplet size with a diameter of approximately 15 μm [38]. After optimizing the spray operating condition, the conductive patterns were finally accurately spray-coated, as shown in Figure 2a.

Compared to inkjet printing, spray coating has an obvious advantage on fabricating accurate patterns. Figure 2a shows the wave-like edge of inkjet-printed patterns, which is mainly attributed to the drop-to-drop distance and component of the solvent. As depicted in Figure 2b, the 10-μm inkjet-printed line is along the 1.5 ~ 3-μm scalloped edge. If the adjacent conductive lines were set closer than 3 μm, the wave-like edge would result in the crosstalk of electrical signal or even worse [25]. Figure 2c reveals a spray-coated silver line with a width of 20 μm, while the edge of the silver line is only 1 μm. It also shows that the edge of the spray-coated line is composed of a mass of silver dots, resulting from the inevitable diffraction of the spraying process. The enlarged view exhibits that the majority of divergent dots are isolated with each other. This indicates that the edge of spray-coated patterns is not conductive, which guarantees the potential of spray-coated silver nanoparticle inks for fabricating accurate patterns in the scale of nanometer.

**Figure 3:**
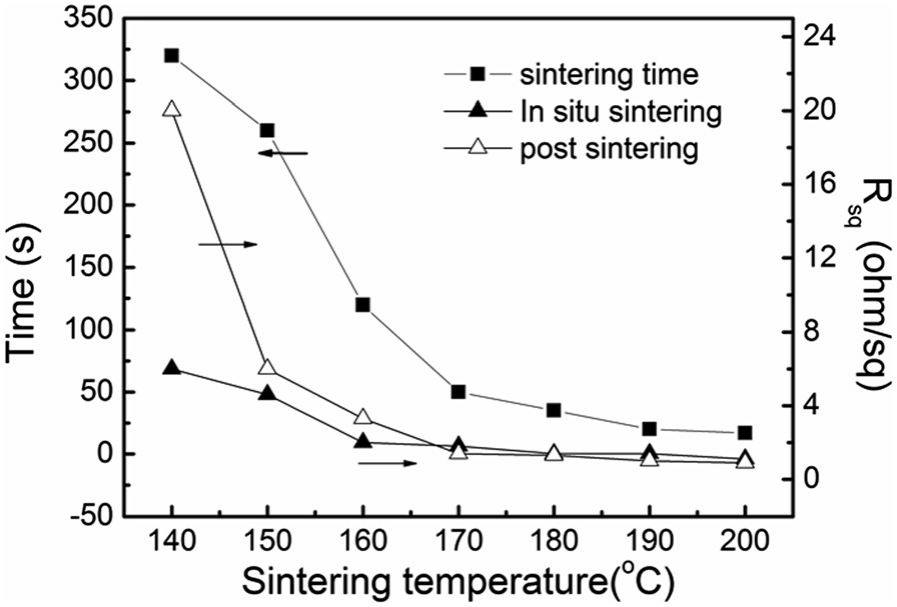
**Parameters of spray-coated silver patterns by post sintering and *in situ *** **sintering process.**

Figure 3 shows the electrical properties of conductive patterns and the relationship between sintering temperature and the time consumption of the sintering process. The transparent ink would turn into black in initial several seconds and then reflect the bulk silver metallic luster after the integrated sintering process. The time consumption of the integrated sintering process declined significantly with the increase of sintering temperature, from 320 s at 140°C to 35 s at 180°C. It indicates that the sintering temperature was the main determinant for obtaining highly conductive patterns by further testing the *R*_sq_, as listed in Table 1. The *R*_sq_ was 20 Ω/cm^2^ at the sintering temperature of 140°C for 320 s, whereas it was significantly decreased to 6 Ω/cm^2^ for 260 s when the temperature was enhanced to 150°C. This lowering tendency of the *R*_sq_ further resulted in a resistance lower than 1 Ω/cm^2^, which was compatible with the requirement for industrial fabrication of conductive circuits [39].

**Table 1:** ***R***_**sq**_**of spray-coated Ag patterns based on various sintering operations**

**Temperature**	***R***_**sq**_	**Time**	***R***_**sq**_	**Time**
**(°C)**	**(post sintering)**	**(post sintering)**	**(*in situ *** **sintering)**	**(*in situ *** **sintering)**
	**(Ω/sq)**	**(s)**	**(Ω/sq)**	**(s)**
140	20.6	320	6.1	52
150	6.3	260	4.6	40
160	3.3	120	2.2	28
170	1.4	50	1.8	20
180	1.2	35	1.4	16
190	1.0	20	1.4	15
200	0.94	17	1.1	15

In order to facilitate the pattern fabrication process to be compatible with the cost-effective fabrication process of printed electronics, an *in situ* sintering process was employed to substitute the general post sintering process. The silver nanoparticle inks were sprayed directly towards the substrate at high temperature (140°C ~ 200°C), in which the drying process of wet droplets and the sintering process of silver nanoparticles took place at the same time. It was shown that a highly conductive pattern with *R*_sq_ of 6 Ω/cm^2^ could be obtained at a low sintering temperature of 140°C, compared to 20 Ω/cm^2^ of the post sintering-processed pattern at the same temperature. More importantly, the time consumption of the *in situ* sintering process to obtain highly conductive patterns at 140°C was significantly reduced to 20 s, which was about one sixth of that of the post sintering process, as listed in Table 1. Meanwhile, the advantages of the *in situ* sintering process on pattern conductivity and time consumption were not further existent when the sintering temperature was higher than 170°C, as shown in Figure 3 and Table 1.

**Figure 4:**
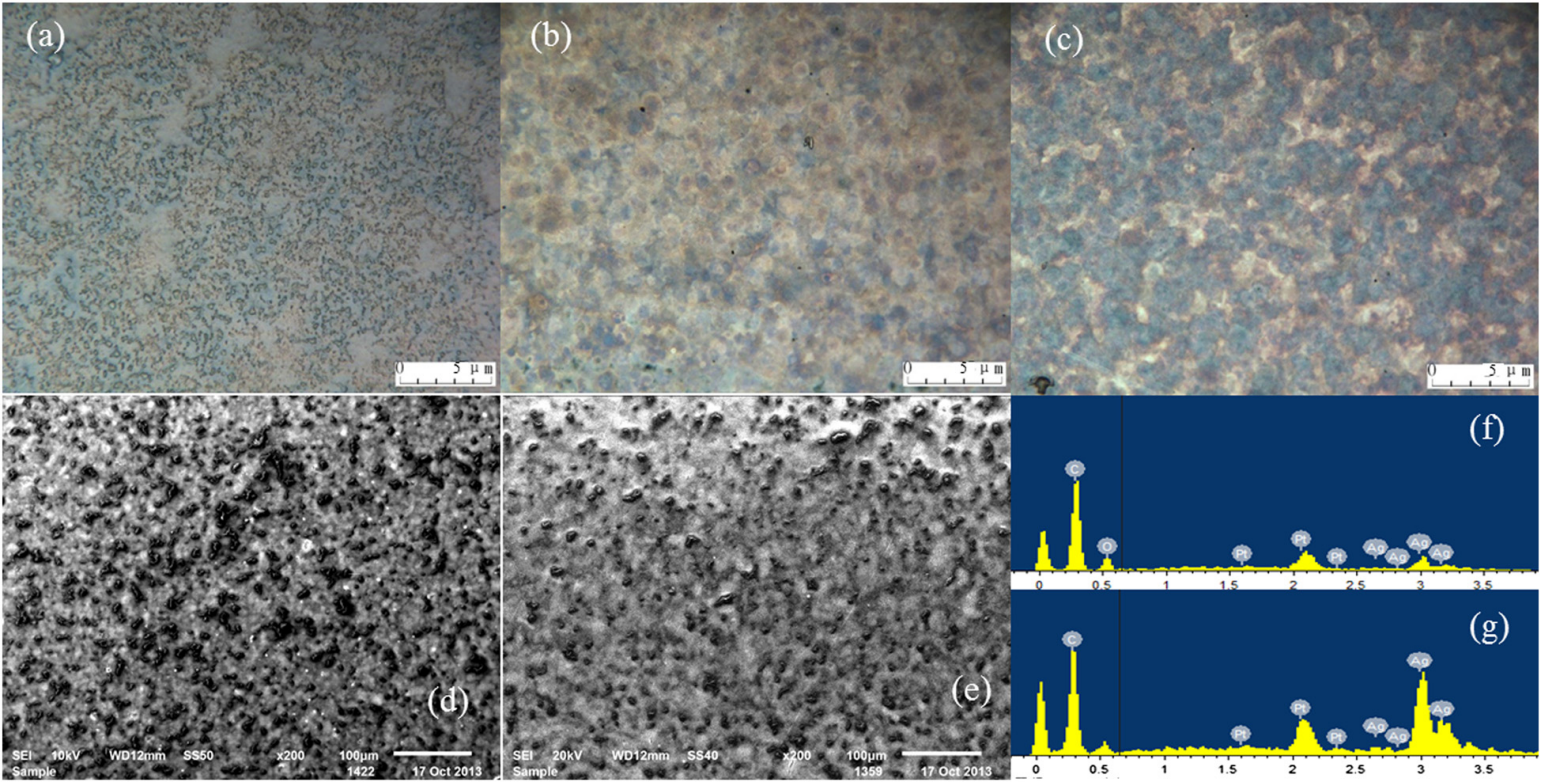
**Metallurgical microscope and SEM images of silver patterns and EDS analysis.** Metallurgical microscope images of silver patterns: **(a)** inkjet-printed and **(b)** spray-coated patterns with 170°C post sintering and **(c)** spray-coated patterns with 170°C *in situ* sintering. SEM images of the morphology of spray-coated silver patterns based on 170°C post sintering **(d)** and *in situ* sintering **(e)** processes. **(f, g)** EDS analysis of the dark bulges and flattened area in (d, e), respectively.

To further illuminate the mechanism of the sintering process of spray-coated silver nanoparticle inks, a metallurgical microscope was used, as shown in Figure 4a,b,c. A general post sintered conductive pattern based on inkjet printing (170°C) is shown in Figure 4a. It can be seen that the silver nanoparticles have melted to integrate to a whole, which reflects the bulk silver metallic luster. However, pores and voids among the nanoparticles are inevitable which limit the conductivity of patterns [40]. Post sintered conductive patterns by spray coating exhibited darker metallic luster compared to the inkjet printed one. It was mainly due to the insufficient evaporation of the stabilizer polymer, as shown in Figure 4b. In this case, the nanoparticles coated by surfactant could hardly break the droplet boundary to form large liquid particles through contacting with each other during the sintering process, resulting in low conductivity. In contrast, as shown in Figure 4c, the *in situ* sintered conductive pattern revealed a continuous silver track with less pores or voids. This was due to the Marangoni flow that facilitated the silver nanoparticles to spread and join large liquid nanoparticles and promote the evaporation of surfactant during the *in situ* sintering process accordingly [41]. In this case, even a low sintering temperature (140°C) could allow the patterns to be conductive with *R*_sq_ of 6 Ω/cm^2^.

Furthermore, SEM was employed to understand the change in the morphology of spray-coated silver nanoparticle inks. Figure 4d,e shows the morphology of spray-coated post sintered and *in situ* sintered conductive patterns, respectively. In Figure 4d, it is obvious that there are a large number of nanoscale dark bulges on the surface of post sintered patterns, and the surface roughness is about 40 nm. However, *in situ* sintered patterns significantly exhibit a lower density of dark bulges. Additionally, *in situ* sintered patterns exhibit a smoother surface with a roughness of 23 nm. Characterized by EDS, a detailed elemental analysis of the sample has been performed. The dark bulges were corresponding to the C element peaking at 0.3 keV. The flat surface was related to the binding energies of Ag *L*_α_ and Ag *L*_β_ at the peaks of 3.0 and 3.2 keV, respectively [42]. The main reason for dense dark bulges in the post sintered pattern was that there was a large space for the stabilizer polymer to transfer to the surface and aggregate to become bulges during sintering at high temperature [41]. In comparison, the relatively sparse dark bulges of the *in situ* sintered pattern can be attributed to the simultaneous evaporation of the stabilizer polymer and sintering of silver inks. Dried droplet limited the mobility of the stabilizer polymer, which was not affected by the latish wet droplet inks. Hence, there were a few dark bulges detected on the surface, but many of them were distributed into the whole pattern vertically. This was also consistent with the lower conductivity of *in situ* sintered conductive patterns at high sintering temperature [40].

**Figure 5:**
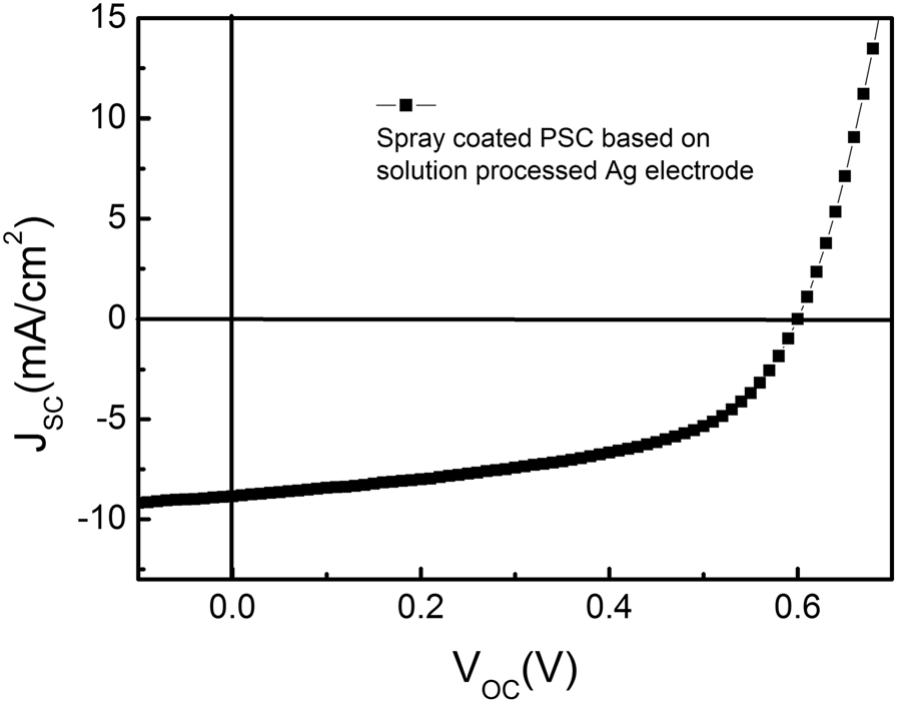
Current density-voltage characteristics of inverted PSC based on spray-coated Ag electrode.

To testify the application of spray-coated silver nanoparticle inks for optoelectronic application, an inverted PSC was fabricated. Figure 5 shows the current density-voltage (*J-V*) characteristics of inverted PSC based on a spray-coated Ag anode, with a current density (*J*_SC_), open-circuit voltage (*V*_OC_), and fill factor (FF) of 8.8 mA/cm^2^, 0.6 V, and 52%, respectively. As listed in Table 2, it can also be observed that the *J*_SC_ and *V*_OC_ were on the same order as those of the devices based on the evaporated Ag anode [24]. However, the FF was significantly lower than that of the general inverted PSC based on the evaporated Ag anode, which was about 60%. It may be attributed to the high temperature of the sintering process at about 160°C ~ 180°C that could damage the active layer materials, resulting in discontinuous paths for charge transportation [43]. Therefore, further work would be focused on reducing the sintering temperature of spray-coated silver nanoparticle inks to obtain high-efficiency PSC.

**Table 2:** Device characteristics of spray-coated PSCs

**Ag electrode**	***∆T *** **(°C)**	**Temperature (°C)**	***V***_**OC **_**(V)**	***J***_**SC **_**(mA/cm**^**2**^**)**	**FF (%)**	**PCE (%)**
*In situ* sintering	135	160	0.60	8.85	52	2.76
Evaporation	-	-	0.59	10.90	60	3.87

## Conclusions

In conclusion, spray coating method was successfully applied for the fabrication of accurate nanoscale conductive patterns consisting of silver nanoparticle inks. Homogeneous and highly conductive patterns with low *R*_sq_ less than 1 Ω/cm^2^ were obtained by optimizing the spray coating parameters. Meanwhile, *in situ* sintering was incorporated to facilitate the sintering process, leading to less time consumption and lower energy cost compared to the general post sintering process. Finally, the potential of silver nanoparticle inks for printed electronics was also testified by fabricating an inverted PSC based on the spray-coated silver electrode, which exhibited a high PCE of 2.76%. This approach would be significantly beneficial to widen the application of silver nanoparticle inks and facilitate it to match the cost-effective and large-scale fabrication process of printed electronics.

## Competing interests

The authors declare that they have no competing interests.

## Authors’ contributions

YZ carried out the design of the experiment and characterization and acquisition of data. SL and WS mainly made contribution on performing the experiment and data analysis. JY is the supervisor of YZ, who is the corresponding author of this work. All authors read and approved the final manuscript.
